# Covid-19 Misinformation Alert, or: “Wash Your Hands and Eat Your Veggies!”

**DOI:** 10.3389/ftox.2020.00004

**Published:** 2020-08-28

**Authors:** Bengt Fadeel

**Affiliations:** Institute of Environmental Medicine, Karolinska Institutet, Stockholm, Sweden

**Keywords:** COVID-19, research ethics, pandemic, preprints, retractions

Since the dawn of the Covid-19 pandemic, an unprecedented number of scientific papers have been published. However, the recent retraction of two high-profile coronavirus papers over data integrity issues has provided a reminder that bad science is omnipresent and may rear its ugly head especially in these pandemic times (Mehra et al., 2020a,b). Obviously, journal editors and reviewers (meaning, all of us) have an important role to play, but as recently pointed out by others, the deluge of publications during the first few months of the current pandemic has already resulted in editor and reviewer fatigue (Rzymski et al., 2020). The situation is compounded by the fact that preprint servers are making vast numbers of papers available to the academic community prior to any peer review (King, 2020; Vabret et al., 2020). Indeed, it is nearly impossible to keep up with this pandemic pace of publishing. However, science has a key role in tackling ongoing and future pandemics, and this is not the time for research exceptionalism; we need to “triage out low-value research” (London and Kimmelman, 2020) and remind ourselves and our students and post docs of the core principles of the scientific method. Naturally, scientists are not immune to the lockdown and its impact on society, and it is understandable that preliminary reports on the virus and the disease were interspersed with personal musings or reflections on lessons learned during the first few months of this global crisis (Kostarelos, 2020; Remsey-Semmelweis et al., 2020). Some authors may also feel the urge to publish in order to further their own bibliometric record. However, the problem that I would like to address here is one of shoddy science and unsound advice based on a disregard for basic scientific principles. Upon a cursory review of the recent Covid-19 literature, it appears that one problem is that some authors do not make the distinction between correlation and causation (Sies, 1988).

In a recent paper, the authors found that there is “a clear interrelated prevalence” between the total number of Covid-19 cases *per* million inhabitants and the gram of spice supply *per* capita *per* day (based on data procured in 2013) (Elsayed and Khan, 2020). Thus, in countries with a lower consumption of spices, a greater number of Covid-19 cases were recorded. The authors concluded that “this is not surprising as herbs and spices are well-known to boost immunity.” Furthermore, they stated that this led them to hypothesize that “spice consumption plays a role in our ability to fight COVID-19,” and they called for research to determine “the translational value” of these findings (Elsayed and Khan, 2020). The problem is that this is the very definition of a circular argument. There are numerous other examples in the literature of well-meaning advice regarding nutritional status and the importance of a balanced diet, but does this really merit further research, or should research efforts be focused on developing an effective vaccine? In some cases, such claims are seemingly innocuous while in other cases, authors draw misleading or unfounded conclusions regarding underlying risk factors and/or potential therapies for Covid-19. For instance, in a recent viewpoint article, Polonikov (2020) described 4 individuals who had contracted Covid-19, for whom a correlation was suggested between glutathione deficiency and moderate or severe disease. Hence, restoration of glutathione levels in Covid-19 patients was suggested as a promising approach for the management of SARS-CoV-2 infection (Polonikov, 2020). The title of the paper goes one step further suggesting “endogenous deficiency of glutathione as the most likely cause of serious manifestations and death in COVID-19 patients.” Clearly, this is not acceptable. There is a huge difference between speculations regarding the mechanism(s) of a disease and its probable cure and touting novel therapies for Covid-19 based on anecdotal evidence; some of the proposed remedies are probably harmless, but some may bring about significant side-effects. The recent chloroquine debacle is a case in point. In another remarkable example, the authors hypothesized that “it may be possible to look in the gut for a solution to or mitigation of SARS-CoV-2 infection” (Kalantar-Zadeh et al., 2020). The authors went on to suggest that an essential step in tackling the disease is “identifying the main gut microbiome species interacting with this virus.” However, even though SARS-CoV-2 may potentially infect ACE2-positive cells lining the gut, it remains unclear why and how the virus would “interact” with bacteria residing in the gut. The authors also hastened to add: “As general advice, frequent snacking between meals may cause dysbiosis and so should be kept to a minimum and only constitute fruit and vegetables, if required” (Kalantar-Zadeh et al., 2020). Finally, in the very last paragraph, the authors invoked nanotechnology, arguing that it may fulfill a critical role in the diagnosis, monitoring, and in the design of effective therapeutic actions for Covid-19 “with relevance for the gut.” However, one cannot help but wonder: do we need nanotechnology, or should we simply eat our veggies? Furthermore, are we really in a position to suggest that “following a healthy and more diverse diet is a logical approach to mitigate the severity of this viral infection as a plausible preventive action?” I find such advice most unhelpful in the face of the seriousness of the ongoing pandemic. We simply do not know enough about SARS-CoV-2 and Covid-19 at this time. Besides, such recommendations, disseminated through reputable journals, may lead to a disregard for other mitigation measures including the wearing of face masks (Zhang et al., 2020).

Smoking (or nicotine exposure, to be more precise) is another example of a contentious topic that seems to side-step the real issues in relation to Covid-19. Hence, while early reports implied that smoking is a predisposing risk factor for Covid-19 disease severity and mortality (Olds and Kabbani, 2020), papers have now emerged claiming that Covid-19 patients who smoke “are more often asymptomatic or exhibit less severe respiratory symptoms than non-smokers,” and that stimulation of acetylcholine receptors on macrophages may inhibit pro-inflammatory cytokines (Kloc et al., 2020). I agree that we should discuss risk factors or factors that could potentially mitigate severe respiratory symptoms in Covid-19 patients, based upon the available evidence. However, I wonder whether we need to remind ourselves of the detrimental effects of smoking on pulmonary and cardiovascular health? Do we really want to fight fire with fire? Moreover, as pointed out by the World Health Organization, studies addressing smoking in hospitalized patients are fraught with limitations and population-based studies are needed to address this issue (https://www.who.int/news-room/commentaries/detail/smoking-and-covid-19).

The current coronavirus pandemic is a serious matter and we don't need “armchair” science. Instead, what we need are well-controlled clinical trials of novel or repurposed therapies and vaccines.

## Disclosure

I was trained in virology before switching to toxicology and my laboratory has been engaged in nanosafety research for more than a decade; more recently, we have shifted toward nanomedicine.

## Author Contributions

BF handled the entirety of this paper. The paper reflects the personal opinions of the author.

## Conflict of Interest

The author declares that the research was conducted in the absence of any commercial or financial relationships that could be construed as a potential conflict of interest.
